# Next-generation sequencing based detection of *BRCA1* and *BRCA2* large genomic rearrangements in Chinese cancer patients

**DOI:** 10.3389/fonc.2022.898916

**Published:** 2022-09-06

**Authors:** Dingchao Hua, Qiuhong Tian, Xue Wang, Ting Bei, Lina Cui, Bei Zhang, Celimuge Bao, Yuezong Bai, Xiaochen Zhao, Peng Yuan

**Affiliations:** ^1^ Department of Medical Affairs, 3D Medicines Inc., Shanghai, China; ^2^ Department of Medical Oncology, First Affiliated Hospital of Nanchang University, Nanchang, China; ^3^ National Cancer Center/National Clinical Research Center for Cancer/Cancer Hospital, Chinese Academy of Medical Sciences and Peking Union Medical College, Beijing, China; ^4^ Department of Gastrointestinal Oncology, Key Laboratory of Carcinogenesis and Translational Research, Ministry of Education, Peking University Cancer Hospital & Institute, Beijing, China

**Keywords:** BRCA, large genomic rearrangements, breast cancer, ovarian cancer, pan-cancer, next-generation sequencing

## Abstract

*BRCA1/2* mutation is a biomarker for guiding multiple solid tumor treatment. However, the prevalence of *BRCA1/2* large genomic rearrangements (LGRs) in Chinese cancer patients has not been well revealed partially due to technical difficulties in LGR detection. This study utilized next-generation sequencing (NGS) to analyze the *BRCA1/2* mutation profile, including LGR, in 56126 Chinese cancer patients. We also reported that two ovarian and breast cancer patients with NGS-determined *BRCA1/2* LGR benefited from PARP inhibitors (PARPi). DNA sequencing identified *BRCA1/2* variants (including LGR, pathogenic and likely-pathogenic variants) in 2108 individuals. Seventy patients were discovered to harbor germline LGRs in *BRCA1* and 14 had germline LGRs in *BRCA2*. Among the LGRs detected, exon 1-2 deletion was the predominant LGR (14/70) in *BRCA1*, and exon 22-24 deletion was the most frequent LGR (3/14) in *BRCA2*. Notably, the *BRCA1* exon 7 deletion was a novel LGR and was identified in six patients, suggesting a specific LGR in Chinese cancer patients. The prevalence analysis of *BRCA1* and *BRCA2* LGRs across multiple cancers revealed that *BRCA1* LGR more frequently occurred in ovarian cancer (1.31%, 33/2526), and *BRCA2* LGR was more commonly seen in cholangiocarcinoma (0.47%, 2/425). Two ovarian and breast cancer patients with *BRCA1/2* LGR benefited from PARPi therapy. This is the first study to reveal the *BRCA1/2* LGR profile of a Chinese pan-cancer cohort by using an NGS-based assay. Two breast and ovarian cancer patients harboring NGS-determined *BRCA1/2* LGR benefited from PARPi, indicating that NGS-based detection of *BRCA1/2* LGR has the potential to guide PARPi treatment.

## Introduction

*BRCA1* and *BRCA2* are tumor suppressor genes that protect genomic stability *via* homologous recombination repair (HRR). Germline mutations in *BRCA1*/*2* are associated with a high risk of cancers, particularly breast and ovarian cancers. PARP inhibitors (PARPi), such as olaparib and niraparib, induce synthetic lethality in tumors with HRR-deficiency and are currently available in the clinic (1, 2). Accurately characterizing *BRCA1/2* mutational status, including *BRCA1/2* somatic and germline mutations, can guide treatment for patients with these gene alterations and help assess the risk of developing hereditary breast and ovarian cancer (HBOC). Different from the well-studied single nucleotide variations (SNVs) and small insertions or deletions (InDels) of *BRCA1/2* genes, large genomic rearrangements (LGRs) refer to the genomic reconstruction of large DNA fragments that affect one or more exons. Most of these chromosomal changes are pathogenic. In particular, *BRCA1/2* germline LGR can be inherited, which may lead to an accumulation of HBOC in a familial manner (3). Thus, accurate characterization of LGR is of great clinical significance.

Indeed, LGR cannot be well identified using conventional methods but can be detected with copy-number sensitive assays such as multiplex ligation-dependent probe amplification (MLPA) and quantitative multiplex PCR of short fluorescent fragments (QMPSF). Their shared limitations are the requirements of high-purity DNA samples and high-order multiplex PCR to guarantee wide coverage. The next-generation sequencing (NGS)-based method is a newcomer with high-throughput that has the potential to detect various types of mutations in a one-set test (4). Given the increasing awareness of LGR significance, the LGR profile of different racial populations has been studied in recent years. According to data from Caucasian populations, LGRs account for approximately 4%-28% of *BRCA1/2* mutations and 10% of *BRCA1/2* germline mutations (4, 5). Partially due to the technical difficulties in detecting LGR, the profile of *BRCA1/2* LGR in Asian cancer patients has not been well elucidated (6–8). Data from the Chinese mainland are extremely limited (9, 10). This study deciphered the *BRCA1/2* variant profile, including LGR, in a large population of 56126 pan-cancer patients from the Chinese mainland; the largest Chinese cohort analyzed using NGS for *BRCA1/2* LGR cancers to date. We also explored the utility of NGS-based detection of *BRCA1/2* LGR in guiding the treatment of breast and ovarian cancer patients.

## Method

### Data source and patients

We analyzed the sequencing data of tumor specimens from 56126 pan-cancer patients in the 3DMed database. All breast cancer and ovarian cancer patients included in this study had both FFPE tissues/plasma samples and paired white blood cell samples for screening paired somatic and germline variants in *BRCA1/2*. Ovarian and breast cancer patients reported in the case presentation section were identified according to their electronic medical records at First Affiliated Hospital of Nanchang University and National Cancer Center/National Clinical Research Center for Cancer/Cancer Hospital. This study was approved by each site’s ethics committee.

### Sample preparation and next-generation sequencing

Formalin-fixed paraffin-embedded (FFPE) tissue or blood samples were obtained from patients and subjected to somatic mutation profiling using NGS. Paired white blood cells were also collected to identify germline mutations. NGS was performed at 3D Medicines Inc., a College of American Pathologists (CAP) and Clinical Laboratory Improvement Amendments (CLIA)-certified laboratory. The sequencing coverage and quality statistics of each sample are summarized in **Supplementary Table S1**. The details of sample processing, library preparation, and sequencing data analysis are shown in the **Supplementary Methods**. Pathogenic and likely-pathogenic germline variants were identified according to the American College of Medical Genetics and Genomics (ACMG)/Association for Molecular Pathology (AMP) guidelines (11–13). Pathogenic and likely-pathogenic somatic variants were identified as per AMP/American Society of Clinical Oncology (ASCO)/College of American Pathologists (CAP) guidelines (14).

### Development of the LGR detection assay

Germline LGRs in *BRCA1* and *BRCA2* were detected using a capture-based NGS method developed by 3D Medicines Inc. The details of LGR detection method have been submitted for a Chinese patent (Patent NO. CN111534579A; https://pss-system.cponline.cnipa.gov.cn/). The capture probes of the invention comprise exon probes and single nucleotide polymorphism (SNP) probes. The exon probes comprise at least three probes aiming at each exon of the targeted gene and 60 bp regions at two ends of the exon. The SNP probes comprise SNP loci with a population occurrence frequency of 30%-70% in the gene intron region and the upstream and downstream within the 5000 bp ranges of the gene. Meanwhile, the invention further provides an algorithm formed by integrating a read depth algorithm module, an allelic imbalance algorithm module, and a discordant sequence algorithm module. The algorithm is used for carrying out large genomic rearrangement analyses on the NGS sequencing data. False positives caused by single-base variation were reduced, and high detection performance was achieved for exon amplification. (**Supplementary Figures S1, S2**). A total of 38 patients with solid tumors who had FFPE tissues/plasma samples and paired white blood cell samples were included for technical validation. NGS was performed on FFPE tissues/plasma samples and paired white blood cell samples, and MLPA, the gold standard for LGR detection, was performed on white blood cell samples. The NGS-based LGR detection assay had 100% concordance with MLPA in detecting germline LGRs (**Supplementary Tables S2, S3**).

## Results

### Sequencing data of tumor samples from Chinese cancer patients

A total of 56126 cancer patients were screened for variants in *BRCA1/2* genes using NGS. DNA sequencing analysis identified *BRCA1/2* variants (including pathogenic and likely pathogenic variants in *BRCA1/2* and germline LGR in *BRCA1/2*) in 2108 individuals (*BRCA1*, n=888; *BRCA2*, n=1284). Sixty-four (64/2108, 3.0%) patients had both *BRCA1* and *BRCA2* mutations. Seventy patients were discovered to harbor germline LGRs in *BRCA1* and 14 in *BRCA2*. Both in ovarian cancer and breast cancer, the *BRCA1/2* germline variant was more frequently observed than the *BRCA1/2* somatic variant (ovarian cancer, *BRCA1/2* germline, 369/2526, 14.6%; *BRCA1/2* somatic, 127/2526, 5.0%; breast cancer, *BRCA1/2* germline, 170/1788, 9.5%; *BRCA1/2* somatic, 43/1788, 2.4%), which were comparable to the findings of previous studies (15–18). The prevalence of *BRCA1/2* variants in different cancers is summarized in **Supplementary Tables S4–S6**.

### Characterization of BRCA1/2 LGR

The median age of patients with *BRCA1* and *BRCA2* LGR was 54 and 63 years, respectively. Female was the predominant subpopulation in patients with *BRCA1* LGR and *BRCA2* LGR. No significant difference was found in age or sex distribution between patients with *BRCA1* LGR and *BRCA2* LGR (**Supplementary Table 7**).

**Figure 1:**
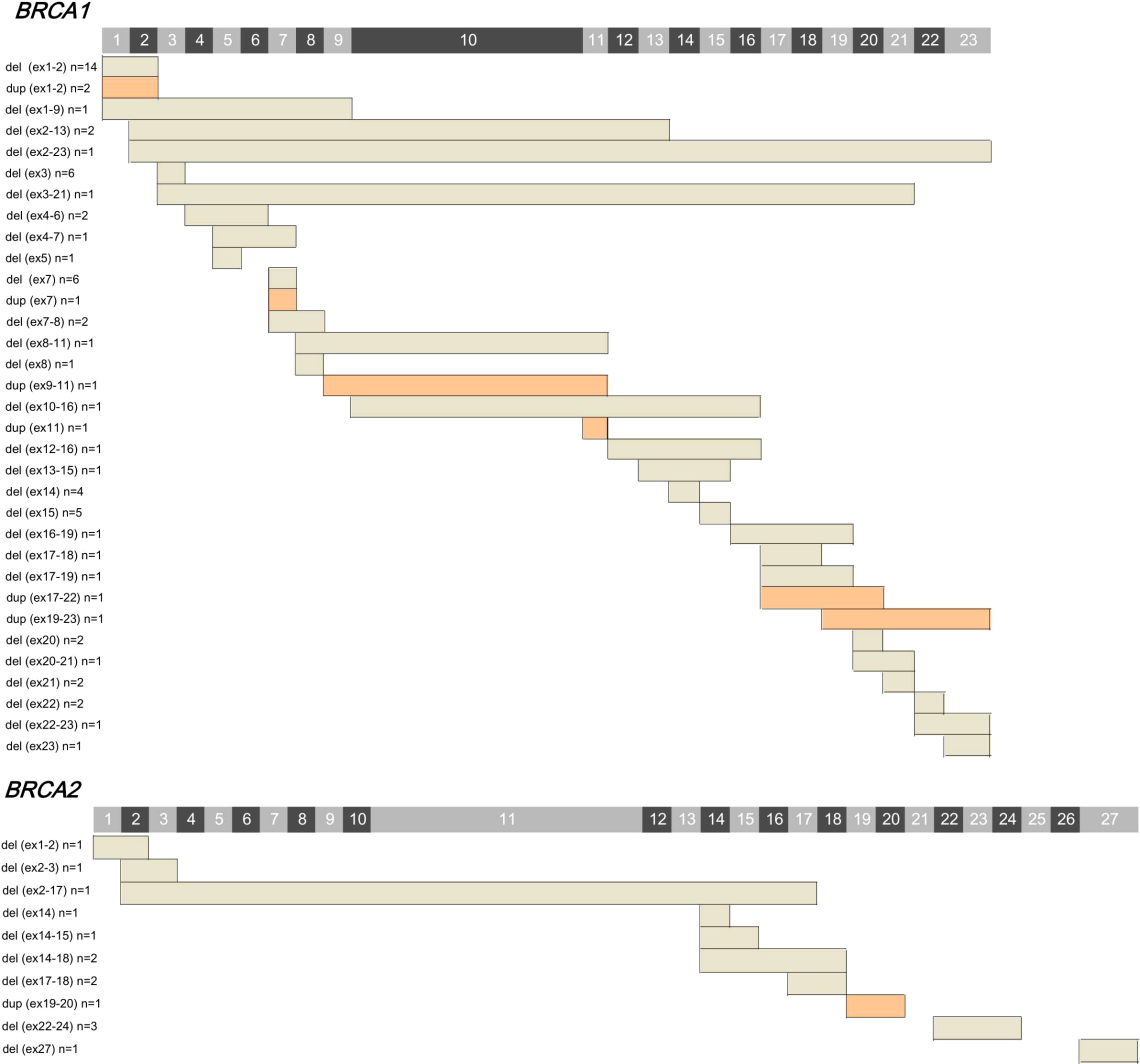
The spectrum of large genetic rearrangements (LGRs) detected in *BRCA1* and *BRCA2* genes. Exon numbers are indicated on the top. The characteristics of the detected LGRs are listed on the left. The affected exons are shown as bars, among which, grey represents deletion, and orange indicates duplication. Actual locations of breakpoint are not implied.

Among the 84 LGRs detected in *BRCA1/2*, exon 1-2 deletion was the predominant mutation type (20.0%, 14/70), followed by exon 3 deletion (8.6%, 6/70), exon 7 deletion (8.6%, 6/70), exon 15 deletion (7.1%, 5/70), and exon 14 deletion (5.7%, 4/70). Among the LGRs identified in *BRCA2*, exon 22-24 deletion was more common (21.4%, 3/14) (**Figure 1**). A total of 43 identical LGRs were identified, of which six were novel, and the remaining 37 have been previously reported. Exon 7 deletion in *BRCA1* was identified in six patients and was not reported previously in cohorts from other countries, which indicated a novel or specific LGR type in Chinese cancer patients. Five of 6 cases who had exon 7 deletion in *BRCA1* were subjected to MLPA test, and all five cases were turned out to be *BRCA1* LGR-positive by MLPA. In addition, exon 10−16 deletion (n=1) in *BRCA1* and exon 2−3 (n=1), exon 2−17 (n=1), exon 14 (n=1), and exon 27 (n=1) deletions in *BRCA2* were other novel LGRs. Deletion was the primary LGR subtype among both *BRCA1* (deletion. vs. duplication; 90.0% vs. 10.0%) and *BRCA2* LGRs (deletion. vs. duplication. 92.9% vs. 7.1%) (**Supplementary Table S8**). The characteristics of *BRCA1* LGR across multiple cancers are summarized (**Supplementary Table S9, Table S10**).

**Figure 2:**
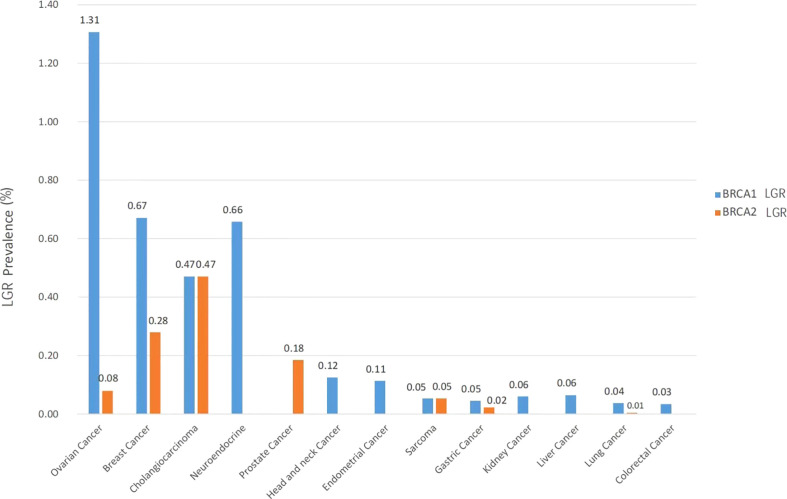
Prevalence of *BRCA1* (orange) and *BRCA2* (blue) LGR across multiple cancer types. Cancer types included ovarian cancer (n = 2526), breast cancer (n = 1788), cholangiocarcinoma (n = 425), neuroendocrine (n = 152), prostate cancer (n = 1083), head and neck cancer (n = 801), endometrial cancer (n = 880), sarcoma (n = 1863), gastric cancer (n = 4427), kidney cancer (n = 1545), liver cancer (n = 4973), lung cancer (n = 23920) and colorectal cancer (n = 11743).

The prevalence of *BRCA1* and *BRCA2* germline LGRs across multiple cancer types is shown in **Figure 2**. *BRCA1* LGR more frequently occurred in ovarian cancer (1.31%, 33/2526), followed by breast cancer (0.67%, 12/1788) and neuroendocrine cancer (0.66%, 1/152). *BRCA2* LGR more commonly occurred in cholangiocarcinoma (0.47%, 2/425), breast cancer (0.28%, 5/1788), and prostate cancer (0.18%, 2/1083) (**Figure 2**). Among the 70 patients with *BRCA1* LGR, patients with ovarian cancer accounted for the largest proportion (33/70, 47.1%), followed by breast cancer (12/70, 17.1%) and lung cancer (9/70, 12.9%). Of the 14 patients with *BRCA2* LGR, 5 (5/14, 35.7%) had breast cancers, and two had ovarian cancers (2/14, 14.3%) (**Supplementary Table S11**).

### Ovarian cancer case presentation

**Figure 3:**
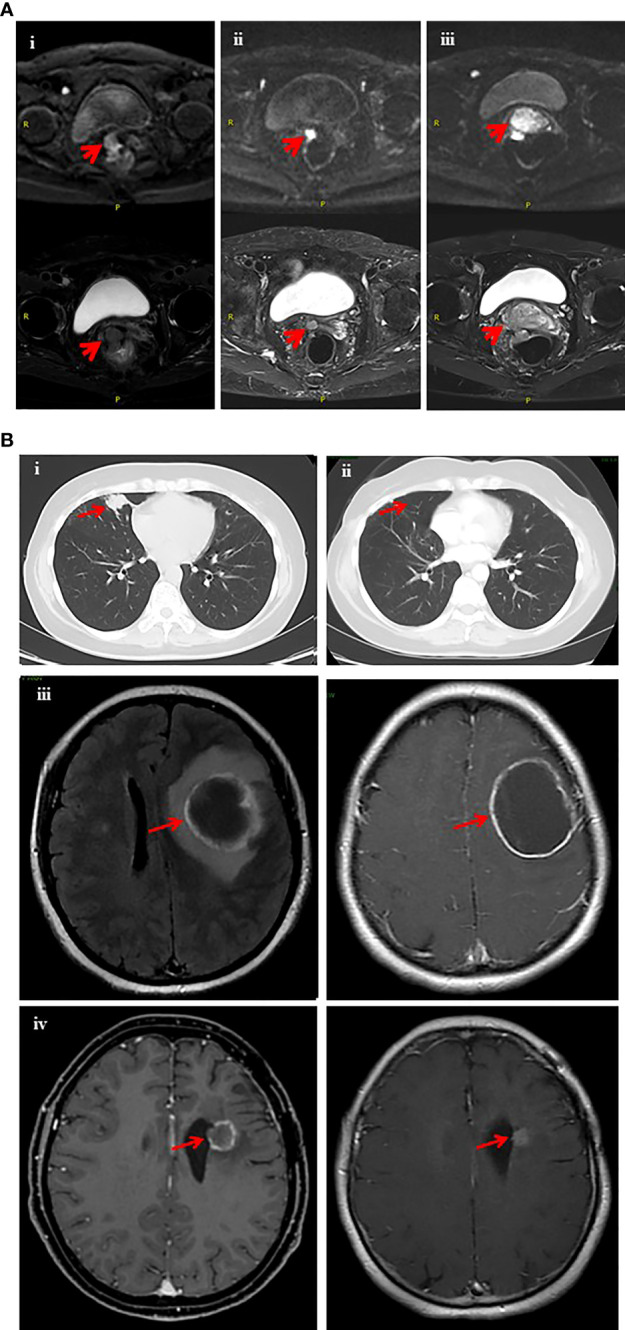
Imaging records of two patients who had *BRCA1/2* LGRs and benefited from PARP inhibitors. **(A)** Magnetic resonance imaging (MRI) scan of the pelvic before and after fluzoparib treatment (ovarian cancer case). The red arrows indicate tumor lesions. **(i)** The pelvic MRI scan on March 5, 2021 revealed tumor status before fluzoparib treatment (5.5*3.4 cm). **(ii)** Pelvic MRI on June 9, 2021 indicated a significant decrease in tumor size (1.9 *1.6 cm) after fluzoparib treatment. **(iii)** The pelvic MRI scan on August 10, 2021 revealed progression of tumor lesions (3.0*4.2 cm) after 3 months of discontinuation of fluzoparib. **(B)** Computed tomography CT scan of the chest and magnetic resonance imaging (MRI) scan of the brain (breast cancer case). The red arrows indicate tumor lesions. **(i)** Chest CT in May 2017 revealed a metastatic lesion (approximately 2.3*1.8 cm) in the middle lobe of the right lung. **(ii)** The patient received surgery in September 2017 and maintenance therapy with olaparib. Chest CT in October 2021 indicated pulmonary metastasis disappeared during maintenance therapy with olaparib. **(iii)** Brain MRI in August 2017 revealed a metastatic lesion with ring-like enhancement in the left frontal lobe with peri-tumoral edema. **(iv)** Brain MRI in October 2021 indicated a significant shrinkage in brain metastasis lesions during the maintenance therapy with olaparib.

On June 5, 2019, a 69-year-old woman with ovarian cancer underwent cytoreductive surgery consisting of bilateral salpingo-oophorectomy, hysterectomy, omentectomy, partial rectectomy, and removal of adhesion between the ovary and pelvic/peritoneum. She was considered to have FIGO stage II disease according to the pathologic results and findings observed during surgery. The pathological results revealed high-grade serous carcinoma in the right adnexa. Tumor lesions were also involved in the epiploic appendix and the rectum anterior wall. She received six cycles of adjuvant intravenous chemotherapy (120 mg paclitaxel + 50 mg lobaplatin) after surgery. On July 16, 2020, the patient visited our clinic and presented with a high level of CA125 (3775 U/mL) and a high ROMA index. Enhanced computed tomography (CT) revealed multiple nodular soft-tissue density shadows, with obviously non-uniform enhancement. She was considered to have disease recurrence and was therefore treated with adjuvant intravenous chemotherapy (50 mg doxorubicin hydrochloride + 50 mg lobaplatin) for six months. On February 17, 2021, pathological examination of the vaginal stump revealed ovarian-derived high-grade serous cystadenocarcinoma vaginal metastasis. Magnetic resonance imaging (MRI) on March 5, 2021 showed a soft-tissue density mass shadow in the anastomotic stoma of a previous rectal resection, suggesting that the disease relapsed (**Figure 3A**). Molecular profiling of vaginal stump biopsy identified a *BRCA1* germline exon 8-11 deletion. Accordingly, the patient was administered the PARPi fluzoparib, which led to a drastic decrease in CA125 (decreased to a normal level with a reduction of 85%) and a shrinkage in the pelvic lesion (**Figure 3A**), achieving partial response (PR). After six weeks of fluzoparib treatment, the patient experienced a grade 3 decrease in platelet number in peripheral blood, for which she discontinued fluzoparib administration. Upon discontinuation of fluzoparib, MRI revealed an enlarged irregular soft-tissue nodular shadow in the anastomotic stoma of previous rectal resection and a drastic increase in the level of serum CA125, which was nine folds higher than the normal level.

### Breast cancer case presentation

On July 11, 2011, a 40-year-old woman was diagnosed with stage IIA (pT2N0M0) triple-negative breast cancer (HER2-, ER-, and PR-negative). She received chemo-radiotherapy after resection of her right breast mass. In June 2014, a nodule biopsy of the left breast revealed invasive breast carcinoma. The patient underwent extensive resection of the left breast mass and lymph node dissection. Between August 21, 2014 and December 16, 2014, she participated in a clinical trial of paclitaxel liposomes combined with carboplatin (Clinical trial NCT01150513). The patient suffered from grade 3 gastrointestinal reactions and leukocytosis upon six cycles of treatment. The patient had stable disease until metastases occurred on the lung in May 2017 (**Figure 3B**). She underwent right-lung middle lobe resection and mediastinal and hilar lymph node dissection. Immunohistochemical staining of the lung lesion showed negative results for NapsinA, TTF1, ER, PR, HER2, CK5/6 and GATA3, and Ki67 nuclear positivity in 70% stromal cells. The radiotherapist considered the effect of radiotherapy to be poor for intracranial hypertension and obvious peripheral edema. To explore other potential therapeutic opportunities, NGS was performed on the retrieved right lung and left breast tumor lesions collected in July 2017 and June 2014, respectively. Molecular profiling identified potentially pathogenic mutations, including germline exon 2 deletion in *BRCA1* and somatic gene arrangement in *BRCA2*-*STARD13* from the right lung lesion. The patient refused to receive chemotherapy, and according to the NGS results she started to receive the PARPi olaparib at a dose of 200 mg twice per day on September 20, 2017. Disease had remained stable as of the time of the submission of this manuscript (**Figure 3B**).

## Discussion

In clinical practice, MLPA is the most widely used method to detect *BRCA1/2* LGRs (19, 20). The primary weaknesses of MLPA are as follows: a high requirement for the purity of nucleic acid samples, due to which alcohol, metal ions, phenol, and TRIzol all affect the performance of MLPA; false-positive result tends to happen when SNV occurs at the probe binding site; poor performance to detect duplication. A wide range of other methods are available for LGR detection, but no one has overwhelming advantages. Southern blotting, real-time PCR, dual-color fluorescence *in situ* hybridization (FISH), comparative genomic hybridization, and multiplex amplifiable quantification all have disadvantages such as being time-consuming, having a high false-positive rate, and having low sensitivity (21). In addition, *BRCA1/2* has a long gene sequence and multiple pathogenic mutation types, including missense, frameshift, nonsense, and LGR, rendering it challenging to comprehensively detect *BRCA1/2* mutations. Herein, we utilized a capture-based NGS method to reveal the profile of *BRCA1/2* mutation, making it accurate and time-saving to characterize point mutations and small or large insert-deletions in a one-set test. The well-designed and strictly validated NGS method has full coverage for all exons and sufficient sequencing depth, demonstrating 100% concordance with MLPA.

Ovarian and breast cancers are the leading causes of gynecologic cancer-related death. *BRCA1/2-*mutated cancer patients represent a typical molecular subset. For ovarian cancer patients, germline and somatic *BRCA1/2* mutations have been utilized as biomarkers for the use of PAPRi (1). For breast cancers, National Comprehensive Cancer Network (NCCN) guidelines recommend that recurrent or metastatic patients with germline *BRCA1/2* mutations receive PARPi therapy (2). In this study, we presented two cases with *BRCA1/2* LGR as determined by NGS (ovarian cancer case: *BRCA1* germline exon 8-11 deletion; breast cancer case: *BRCA1* germline exon 2 deletion), and both patients benefited from PARPi therapy, providing evidence for the feasibility and significance of NGS-based LGR detection in guiding treatment. Further studies are warranted to confirm the findings.

In this study, the frequency of co-occurring variants in both the *BRCA1* and *BRCA2* genes in the pan-cancer cohort was approximately 3%, which was somehow higher than that of most previous studies (22–25). The possible reason for the relatively high frequency in our study might be the fact that except for pathogenic variants, we also included likely-pathogenic variants and LGRs, which were not included in some studies. Another possibility was that we analyzed the sequencing data in a pan-tumor cohort, which might have led to the discrepancy among studies.

One of the limitations of this study was that the NGS platform employed in our study could not identify the accurate breakpoints of LGR. Breakpoints frequently occur in introns, the length of which can be thousands of bp. The NGS assay applied for LGR detection was designed with capture probes covering the whole exons and extending 60 bp into intron regions at both ends of every exon, which was incapable of identifying accurate breakpoints in introns. Due to a lack of breakpoint information for these LGRs, predicting the impact of these LGRs on *BRCA1/2* proteins was theoretically not feasible. Based on previous reports, deletions in *BRCA1/2* exons could result in premature termination of the *BRCA1* protein, an in-frame deletion, and prevention of transcription. Rearrangements involving different exons in distinct domains may have distinct effects on protein functions (26).

Most of previous studies regarding *BRCA1/2* LGR have primarily focused on ovarian and breast cancer. This study examined the prevalence of *BRCA1/2* germline LGRs in multiple solid tumors. Of note, the prevalence of *BRCA1/2* germline LGR in other cancers, except for ovarian and breast cancers, might be underestimated because a few cases were screened only with tissue specimens. To the best of our knowledge, this was the first and largest study to depict the *BRCA1/2* LGR profile of Chinese pan-cancer patients by using an NGS-based assay. We also reported two cases who had *BRCA1/2* LGR benefited from PARPi, supporting the feasibility of NGS-based detection of *BRCA1/2* LGRs to guide treatment.

## Data availability statement

The VCF files of 84 LGR cases were uploaded to the GVM database (Accession NO. GVM000365, Project NO. PRJCA010855, https://ngdc.cncb.ac.cn/gvm/). Other datasets generated and/or analyzed during the current study are available from the corresponding author on reasonable request. 

## Ethics statement

The studies involving human participants were reviewed and approved by The ethics committees of the First Affiliated Hospital of Nanchang University and National Cancer Center/National Clinical Research Center for Cancer/Cancer Hospital. The patients/participants provided their written informed consent to participate in this study. Written informed consent was obtained from the individual(s) for the publication of any potentially identifiable images or data included in this article.

## Author contributions

XZ, DH, and PY conceptualized the study; DH, QT, and XW, BZ, YB, and CB designed the Methods; DH, QT, XW, and LC collected, analyzed, and interpreted the patient data regarding the discovery and validation cohorts. TB and DH were major contributors in writing the manuscript. TB, XZ, and PY conducted critical revision of the manuscript for important intellectual content. DH, QT, and XW contributed equally and served as co-first authors. All authors read and approved the final manuscript.

## Funding

This study was funded by the Beijing Hope Run Special Fund of Cancer Foundation of China (LC2019B16 to Xue Wang).

## Acknowledgments

We would like to thank all coordinators at the First Affiliated Hospital of Nanchang University, National Cancer Center/National Clinical Research Center for Cancer/Cancer Hospital, Chinese Academy of Medical Sciences and Peking Union Medical College and 3D Medicines Inc. for supporting this study.

## Conflict of interest

D Hua, T Bei, L Cui, B Zhang, C Bao, Y Bai, and X Zhao are employees of 3D Medicines Inc.

The remaining authors declare that the research was conducted in the absence of any commercial or financial relationships that could be constructed as a potential conflict of interest.

The reviewer (JL) declared a shared affiliation, with no collaboration, with the authors (XW, PY) to the handling editor at the time of review

## Publisher’s note

All claims expressed in this article are solely those of the authors and do not necessarily represent those of their affiliated organizations, or those of the publisher, the editors and the reviewers. Any product that may be evaluated in this article, or claim that may be made by its manufacturer, is not guaranteed or endorsed by the publisher.
