# Supplementary material for: Next-generation sequencing based detection of BRCA1 and BRCA2 large genomic rearrangements in Chinese cancer patients
Source: Front Oncol. 2022 Sep 6;12:898916. doi: 10.3389/fonc.2022.898916 (PMC9487528; doi:10.3389/fonc.2022.898916)
Supplement: Supplementary file 1 [file DataSheet_1.docx]

**Supplementary Figure S1. Data processing for *BRCA1/2* LGR detection**

**
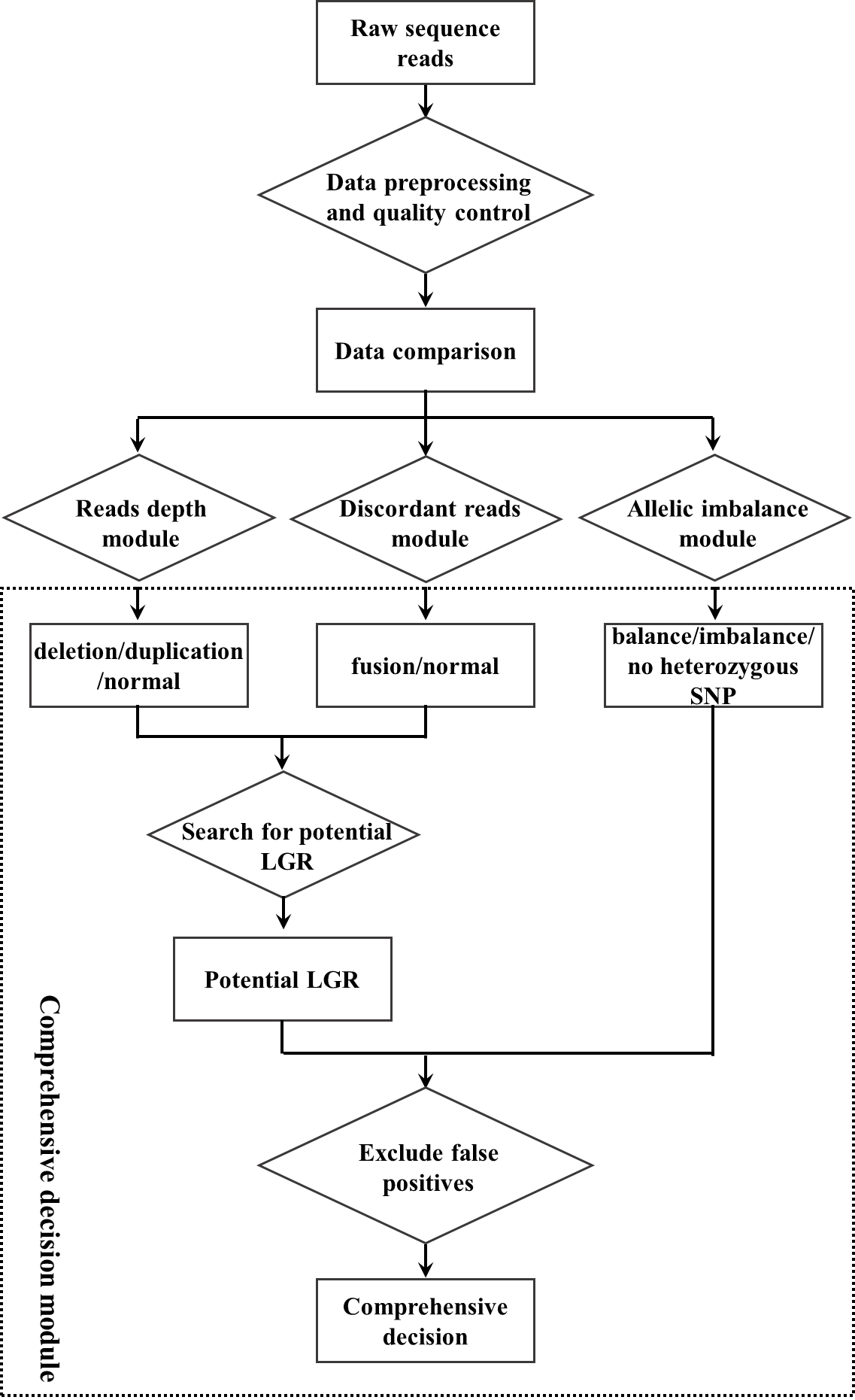
**

**Supplementary Figure S2. Comprehensive decision module for LGR detection**

**
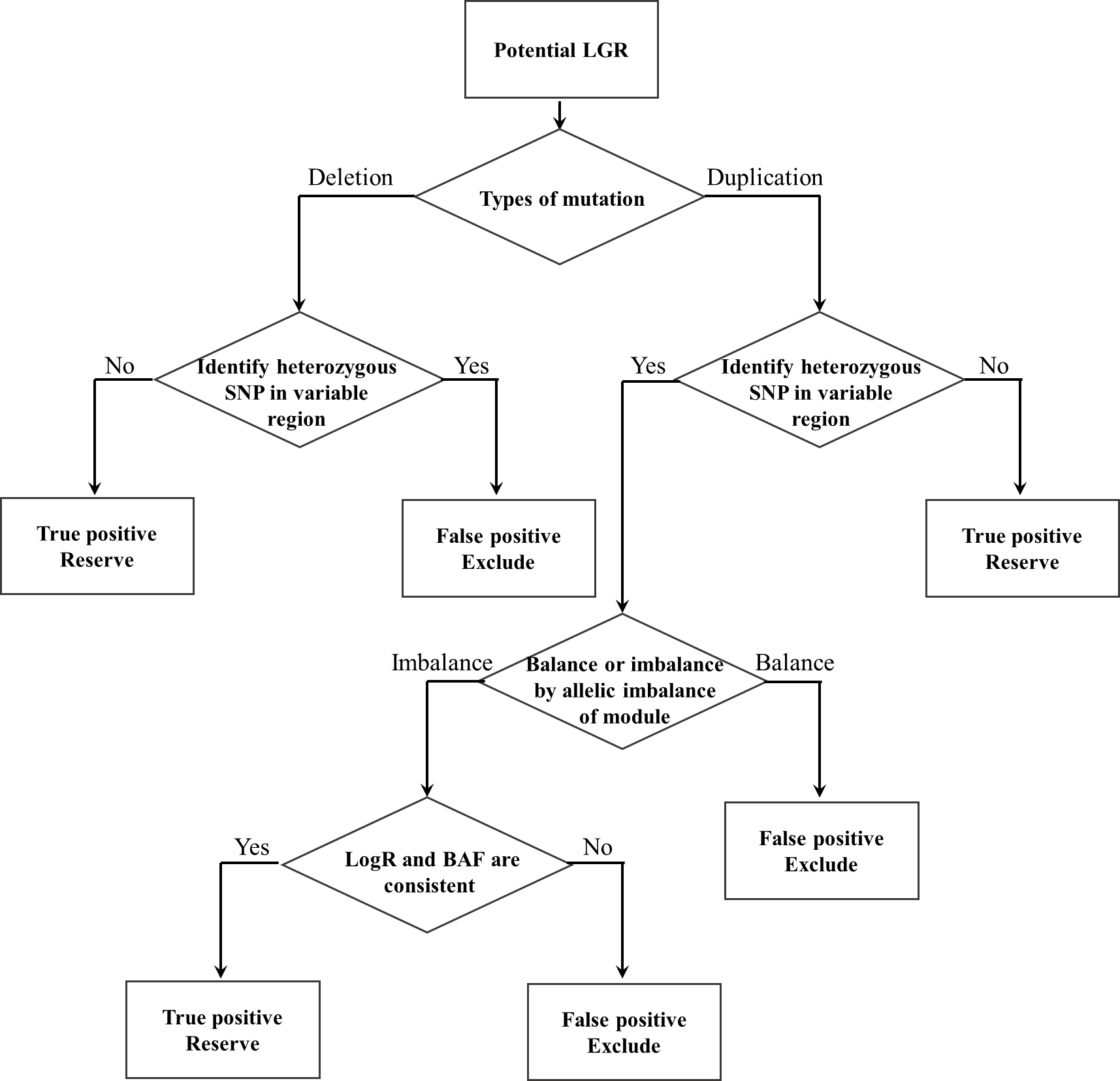
**
