# Supplementary material for: Next-generation sequencing based detection of BRCA1 and BRCA2 large genomic rearrangements in Chinese cancer patients
Source: Front Oncol. 2022 Sep 6;12:898916. doi: 10.3389/fonc.2022.898916 (PMC9487528; doi:10.3389/fonc.2022.898916)
Supplement: Supplementary file 2 [file DataSheet_2.docx]

**Short report: Next-generation sequencing based detection of large *BRCA1* and *BRCA2* genomic rearrangements in Chinese cancer patients**

Dingchao Hua, Qiuhong Tian, Xue Wang, Ting Bei, Lina Cui, Bei Zhang, Celimuge Bao, Jie Wang, Yuezong Bai, Xiaochen Zhao, Peng Yuan

**Supplementary Methods**

***Next-generation sequencing (NGS)***

DNA preparation, sequencing, and data analysis were conducted at a College of American Pathologists (CAP)-accredited and Clinical Laboratory Improvement Amendments (CLIA)-certified laboratory (3D Medicines Inc., Shanghai, China). Tumor samples with a size ≥ 1 mm^3^ and tumor purity ≥ 20% were qualified. DNA was extracted from formalin-fixed paraffin-embedded (FFPE) tissue samples using the ReliaPrep™ FFPE gDNA Miniprep System (Promega, Madison, WI, USA). Blood samples were centrifuged in Streck tubes within 2 hours of collection at 1,600 g at 4°C for 10 minutes. About 5 ml plasma supernatant was transferred to a new 5 ml microfuge tube and centrifuged at 16,000 g at 4 ℃ for 10 minutes to remove residual cells and debris. Supernatant was transferred into a new tube. Cell-free DNA (cfDNA) was extracted using the QiAmp Circulating Nucleic Acid Kit (Qiagen) following the manufacturer’s instructions. Genomic DNA (gDNA) from white blood cells (WBC) was extracted using the QIAamp DNA Mini Kit (Qiagen). DNA was quantified using the Qubit dsDNA HS Assay Kit (Thermo Fisher Scientific, Waltham, MA, USA). DNA extracts (30–200 ng) were sheared to 250-bp fragments and libraries were constructed with the KAPA Hyper Prep Kit (KAPA Biosystems, Wilmington, MA, USA). Indexed libraries were then subjected to probe-based hybridization with a customized NGS panel targeting cancer-related genes (3D Medicines). The captured libraries were loaded onto a NovaSeq 6000 platform (Illumina, San Diego, CA, USA) for 100 bp paired-end sequencing. Burrows-Wheeler Aligner (v0.7.12) was employed to align raw data of paired samples (tissue or plasma and their paired WBC samples) to the reference human genome hg19. Polymerase chain reaction duplicate reads were removed, and sequence metrics were collected using Picard (v1.130) and SAMtools (v1.1.19), respectively. Somatic single-nucleotide variants (SNVs) were detected using an R package developed in-house to execute a variant detection model based on a binomial test. Local realignment was performed to detect indels. Variants were then filtered by their unique supporting read depth, strand bias, and base quality, as previously described [[1](#_ENREF_1)]. All variants were then filtered using an automated false positive filtering pipeline to ensure sensitivity and specificity at an allele frequency (AF) ≥ 1%. Single-nucleotide polymorphisms (SNPs) and indels were annotated by ANNOVAR using the dbSNP (v138), 1000Genome, and ESP6500 databases (population frequency > 0.015). Pathogenic and likely pathogenic variants, including stop-gain, missense, frameshift and non-frameshift insertions and deletions, were kept [[1](#_ENREF_1)]. Large genomic rearrangement were identified as described in the main text. Germline pathogenic and likely pathogenic variants were identified as per the American College of Medical Genetics and Genomics (ACMG)/Association for Molecular Pathology (AMP) guidelines [[2-4](#_ENREF_2)]. Somatic pathogenic and likely pathogenic variants were identified as per AMP/American Society of Clinical Oncology (ASCO)/College of American Pathologists (CAP) guidelines[[5](#_ENREF_5)].

1. Su, D., et al., *High performance of targeted next generation sequencing on variance detection in clinical tumor specimens in comparison with current conventional methods.* J Exp Clin Cancer Res, 2017. **36**(1): p. 121.

2. Richards, S., et al., *Standards and guidelines for the interpretation of sequence variants: a joint consensus recommendation of the American College of Medical Genetics and Genomics and the Association for Molecular Pathology.* Genetics in medicine : official journal of the American College of Medical Genetics, 2015. **17**(5): p. 405-424.

3. Brandt, T., et al., *Adapting ACMG/AMP sequence variant classification guidelines for single-gene copy number variants.* Genetics in Medicine, 2020. **22**(2): p. 336-344.

4. Riggs, E.R., et al., *Technical standards for the interpretation and reporting of constitutional copy-number variants: a joint consensus recommendation of the American College of Medical Genetics and Genomics (ACMG) and the Clinical Genome Resource (ClinGen).* Genetics in Medicine, 2020. **22**(2): p. 245-257.

5. Li, M.M., et al., *Standards and Guidelines for the Interpretation and Reporting of Sequence Variants in Cancer: A Joint Consensus Recommendation of the Association for Molecular Pathology, American Society of Clinical Oncology, and College of American Pathologists.* The Journal of Molecular Diagnostics, 2017. **19**(1): p. 4-23.
