# Supplementary material for: Next-generation sequencing based detection of BRCA1 and BRCA2 large genomic rearrangements in Chinese cancer patients
Source: Front Oncol. 2022 Sep 6;12:898916. doi: 10.3389/fonc.2022.898916 (PMC9487528; doi:10.3389/fonc.2022.898916)
Supplement: Supplementary file 4 [file DataSheet_4.docx]

**Supplementary Table 2 Data of the technical validation cohort**

| **Pts ID** | **NGS** | **MLPA** | **Sex** | **Age, year** | **Tumor type** |
| --- | --- | --- | --- | --- | --- |
| 7115 | *BRCA2*：exon17-18Del | *BRCA2*：exon17-18Del | female | 63 | Cholangiocarcinoma |
| 9071 | *BRCA1*：exon1-2Del | *BRCA1*：exon1-2Del | female | 45 | Ovarian Cancer |
| 9309 | *BRCA1*：exon1-2Del | *BRCA1*：exon1-2Del | female | 46 | Breast Cancer |
| 13194 | normal | normal | female | 63 | Colorectal Cancer |
| 13227 | normal | normal | male | 78 | Gastric Cancer |
| 13645 | *BRCA1*：exon3Del | *BRCA1*：exon3Del | female | 30 | Breast Cancer |
| 14303 | *BRCA1*：exon17-22Dup | *BRCA1*：exon17-22Dup | female | 42 | Breast Cancer |
| 14493 | *BRCA1*：exon14Del | *BRCA1*：exon14Del | female | 28 | Breast Cancer |
| 14504 | *BRCA1*：exon3Del | *BRCA1*：exon3Del | female | 59 | Ovarian Cancer |
| 14849 | *BRCA1*：exon7Dup | *BRCA1*：exon7Dup | female | 28 | Breast Cancer |
| 14960 | normal | normal | male | 25 | Colorectal Cancer |
| 16223 | *BRCA1*：exon9-11Dup | *BRCA1*：exon9-11Dup | female | 35 | Breast Cancer |
| 18485 | *BRCA1*：exon1-2Dup | *BRCA1*：exon1-2Dup | male | 36 | Liver Cancer |
| 25925 | *BRCA2*：exon19-20Dup | *BRCA2*：exon19-20Dup | female | 42 | Breast Cancer |
| 26395 | *BRCA1*：exon19-23Dup | *BRCA1*：exon19-23Dup | male | 78 | Lung Cancer |
| 27665 | *BRCA1*：exon1-2Dup | *BRCA1*：exon1-2Dup | female | 75 | Ovarian Cancer |
| 27666 | *BRCA1*：exon17-18Del | *BRCA1*：exon17-18Del | female | 63 | Ovarian Cancer |
| 31968 | normal | normal | female | 43 | Gastric Cancer |
| 33846 | *BRCA1*：exon22Del | *BRCA1*：exon22Del | female | 60 | Ovarian Cancer |
| 34704 | *BRCA1*：exon7Del | *BRCA1*：exon7Del | female | 44 | Ovarian Cancer |
| 34878 | *BRCA1*：exon4-6Del | *BRCA1*：exon4-6Del | male | 71 | Colorectal Cancer |
| 35285 | *BRCA1*：exon1-9Del | *BRCA1*：exon1-9Del | male | 64 | Gastric Cancer |
| 37225 | normal | normal | female | 43 | normal |
| 37604 | *BRCA1*：exon1-2Del | *BRCA1*：exon1-2Del | male | 65 | Lung Cancer |
| 41574 | *BRCA2*：exon17-18Del | *BRCA2*：exon17-18Del | female | 46 | Breast Cancer |
| 43192 | *BRCA2*：exon2-17Del | *BRCA2*：exon2-17Del | female | 64 | Colorectal Cancer |
| 43816 | *BRCA1*：exon7Del | *BRCA1*：exon7Del | female | 60 | Lung Cancer |
| 45044 | *BRCA1*：exon7Del | *BRCA1*：exon7Del | male | 67 | Lung Cancer |
| 46073 | *BRCA1*：exon8Del | *BRCA1*：exon8Del | female | 69 | Ovarian Cancer |
| 47341 | *BRCA1*：exon7Del | *BRCA1*：exon7Del | female | 49 | Ovarian Cancer |
| 48711 | *BRCA2*：exon20Del | *BRCA2*：exon20Del | male | 73 | Prostate Cancer |
| 54730 | *BRCA1*：exon7Del | *BRCA1*：exon7Del | female | 47 | Endometrial Cancer |
| 54773 | *BRCA1*：exon2Del | *BRCA1*：exon2Del | male | 49 | Cholangiocarcinoma |
| 55313 | *BRCA1*：exon7-8Del | *BRCA1*：exon7-8Del | male | 71 | Colorectal Cancer |
| 55708 | *BRCA2*：exon22-24Del | *BRCA2*：exon22-24Del | male | 60 | Prostate Cancer |
| 55869 | *BRCA1*：exon22Del | *BRCA1*：exon22Del | female | 47 | Ovarian Cancer |
| 57825 | *BRCA2*：exon22-24Del | *BRCA2*：exon22-24Del | female | 51 | Breast Cancer |
| 57889 | *BRCA2*：exon14-18Del | *BRCA2*：exon14-18Del | female | 55 | Ovarian Cancer |

**Note: NGS, next-generation sequencing; MLPA, multiplex ligation-dependent probe amplification; Del., deletion. NGS was performed on FFPE tissues/plasma sample and paired white blood cell sample, and MLPA was performed on white blood cell sample.**

**Supplementary Table 3 The concordance of next-generation sequencing (NGS) with multiplex ligation-dependent probe amplification (MLPA) in detecting *BRCA1/2* LGR**

|  | | MLPA | | Total |
| --- | --- | --- | --- | --- |
|  |  | + | - |  |
| NGS | + | 33 | 0 | 33 |
|  | - | 0 | 5 | 5 |
| Total | | 33 | 5 | 38 |
| PPV=100% | | | | |
| NPV=100% | | | | |
| Concordance=100% | | | | |

**Note: PPV, positive predictive value; NPV, negative predictive value.**

**NGS was performed on FFPE tissues/plasma sample and paired white blood cell sample, and MLPA was performed on white blood cell sample.**
