# Supplementary material for: Next-generation sequencing based detection of BRCA1 and BRCA2 large genomic rearrangements in Chinese cancer patients
Source: Front Oncol. 2022 Sep 6;12:898916. doi: 10.3389/fonc.2022.898916 (PMC9487528; doi:10.3389/fonc.2022.898916)
Supplement: Supplementary file 6 [file DataSheet_6.docx]

**Supplementary Table S7 Characteristics of patients with *BRCA1/2* LGR in the pan-cancer cohort**

| **Characteristic** | **BRCA1 LGR**, N = 70*^1^* | **BRCA2 LGR**, N = 14*^1^* | **p-value***^2^* |
| --- | --- | --- | --- |
| Age | 54 (43, 65) | 63 (53, 65) | 0.054 |
| Sex |  |  | 0.515 |
| Male | 18 (25.7%) | 5 (35.7%) |  |
| Female | 52 (74.3%) | 9 (64.3%) |  |
| *^1^*Median (interquartile range, IQR); n (%)  *^2^* t-test; Fisher's exact test | | | |

| **Characteristic** | **BRCA1 LGR**, N = 70*^1^* | **BRCA2 LGR**, N = 14*^1^* | **p-value***^2^* |
| --- | --- | --- | --- |
| effect |  |  | 1.000 |
| Del | 63 (90.0%) | 13 (92.9%) |  |
| Dup | 7 (10.0%) | 1 (7.1%) |  |
| *^1^*n (%)  *^2^*Fisher's exact test | | | |

**Table S8 Characterization of *BRCA1/2* LGR across multiple cancers**

**Table S9 Characterization of *BRCA1* LGR across multiple cancers**

| **Characteristic** | **Exon1_2**, N = 16*^1^* | **Exon15_15**, N = 5*^1^* | **Exon3_3**, N = 6*^1^* | **Exon7_7**, N = 7*^1^* |
| --- | --- | --- | --- | --- |
| Tumor type |  |  |  |  |
| Breast Cancer | 2(12.5%) | 0 (0%) | 1(16.7%) | 1(14.3%) |
| Cholangiocarcinoma | 1 (6.3%) | 1 (20.0%) | 0 (0%) | 0 (0%) |
| Colorectal Cancer | 1 (6.3%) | 0 (0%) | 0 (0%) | 0 (0%) |
| Endometrial Cancer | 0 (0%) | 0 (0%) | 0 (0%) | 1  (14.3%) |
| Liver Cancer | 1 (6.3%) | 1 (20.0%) | 0 (0%) | 0 (0%) |
| Lung Cancer | 1 (6.3%) | 1 (20.0%) | 1(16.7%) | 2 (28.6%) |
| Neuroendocrine | 0 (0%) | 1 (20.0%) | 0 (0%) | 0 (0%) |
| Ovarian Cancer | 10 (62.5%) | 1 (20.0%) | 4 (66.7%) | 3 (4.9%) |
| *^1^*n (%) | | | | |

Note: *BRCA1* LGR with frequency >=5 is shown.

**Table S10 Characterization of *BRCA1* LGR across multiple cancers**

| **Characteristic** | **Exon1_2**, N = 1*^1^* | **Exon14_14**, N = 1*^1^* | **Exon14_15**, N = 1*^1^* | **Exon14_18**, N = 2*^1^* | **Exon17_18**, N = 2*^1^* | **Exon19_20**, N = 1*^1^* | **Exon2_17**, N = 1*^1^* | **Exon2_3**, N = 1*^1^* | **Exon22_24**, N = 3*^1^* | **Exon27_27**, N = 1*^1^* |
| --- | --- | --- | --- | --- | --- | --- | --- | --- | --- | --- |
| Tumor type |  |  |  |  |  |  |  |  |  |  |
| Breast Cancer | 0 (0%) | 0 (0%) | 0 (0%) | 1 (50%) | 1 (50%) | 1 (100%) | 0 (0%) | 1 (100%) | 1 (33%) | 0 (0%) |
| Cholangiocarcinoma | 0 (0%) | 1 (100%) | 0 (0%) | 0 (0%) | 1 (50%) | 0 (0%) | 0 (0%) | 0 (0%) | 0 (0%) | 0 (0%) |
| Colorectal Cancer | 0 (0%) | 0 (0%) | 0 (0%) | 0 (0%) | 0 (0%) | 0 (0%) | 1 (100%) | 0 (0%) | 0 (0%) | 0 (0%) |
| Gastric Cancer | 0 (0%) | 0 (0%) | 0 (0%) | 0 (0%) | 0 (0%) | 0 (0%) | 0 (0%) | 0 (0%) | 1 (33%) | 0 (0%) |
| Ovarian Cancer | 0 (0%) | 0 (0%) | 1 (100%) | 1 (50%) | 0 (0%) | 0 (0%) | 0 (0%) | 0 (0%) | 0 (0%) | 0 (0%) |
| Prostate Cancer | 0 (0%) | 0 (0%) | 0 (0%) | 0 (0%) | 0 (0%) | 0 (0%) | 0 (0%) | 0 (0%) | 1 (33%) | 1 (100%) |
| Sarcoma | 1 (100%) | 0 (0%) | 0 (0%) | 0 (0%) | 0 (0%) | 0 (0%) | 0 (0%) | 0 (0%) | 0 (0%) | 0 (0%) |
| *^1^*n (%) | | | | | | | | | | |

Note: *BRCA2* LGR with frequency >=1 are shown.

**Table S11 Distribution of LGR across multiple cancers**

| **Characteristic** | **BRCA1 LGR**, N = 70*^1^* | **BRCA2 LGR**, N = 14*^1^* |
| --- | --- | --- |
| Tumor type |  |  |
| Breast Cancer | 12 (17.1%) | 5 (35.7%) |
| Cholangiocarcinoma | 2 (2.9%) | 2 (14.3%) |
| Colorectal Cancer | 4 (5.7%) | 1 (7.1%) |
| Endometrial Cancer | 1 (1.4%) | 0 (0%) |
| Gastric Cancer | 2 (2.9%) | 1 (7.1%) |
| Head and neck Cancer | 1 (1.4%) | 0 (0%) |
| Kidney Cancer | 1 (1.4%) | 0 (0%) |
| Liver Cancer | 3 (4.3%) | 0 (0%) |
| Lung Cancer | 9 (12.9%) | 0 (0%) |
| Neuroendocrine | 1 (1.4%) | 0 (0%) |
| Ovarian Cancer | 33 (47.1%) | 2 (14.3%) |
| Prostate Cancer | 0 (0%) | 2 (14.3%) |
| Sarcoma | 1 (1.4%) | 1 (7.1%) |
